# Meditation reduces pain-related neural activity in the anterior cingulate cortex, insula, secondary somatosensory cortex, and thalamus

**DOI:** 10.3389/fpsyg.2014.01489

**Published:** 2014-12-16

**Authors:** Hiroki Nakata, Kiwako Sakamoto, Ryusuke Kakigi

**Affiliations:** ^1^Department of Integrative Physiology, National Institute for Physiological SciencesOkazaki, Japan; ^2^Department of Health Sciences, Faculty of Human Life and Environment, Nara Women’s UniversityNara, Japan

**Keywords:** MEG, EEG, fMRI, pain matrix, Yoga

## Abstract

Recent studies have shown that meditation inhibits or relieves pain perception. To clarify the underlying mechanisms for this phenomenon, neuroimaging methods, such as functional magnetic resonance imaging, and neurophysiological methods, such as magnetoencephalography and electroencephalography, have been used. However, it has been difficult to interpret the results, because there is some paradoxical evidence. For example, some studies reported increased neural responses to pain stimulation during meditation in the anterior cingulate cortex (ACC) and insula, whereas others showed a decrease in these regions. There have been inconsistent findings to date. Moreover, in general, since the activities of the ACC and insula are correlated with pain perception, the increase in neural activities during meditation would be related to the enhancement of pain perception rather than its reduction. These contradictions might directly contribute to the ‘mystery of meditation.’ In this review, we presented previous findings for brain regions during meditation and the anatomical changes that occurred in the brain with long-term meditation training. We then discussed the findings of previous studies that examined pain-related neural activity during meditation. We also described the brain mechanisms responsible for pain relief during meditation, and possible reasons for paradoxical evidence among previous studies. By thoroughly overviewing previous findings, we hypothesized that meditation reduces pain-related neural activity in the ACC, insula, secondary somatosensory cortex, and thalamus. We suggest that the characteristics of the modulation of this activity may depend on the kind of meditation and/or number of years of experience of meditation, which were associated with paradoxical findings among previous studies that investigated pain-related neural activities during meditation.

## INTRODUCTION

Some highly trained meditators reported that they did not feel pain during meditation, and in an attempt to demonstrate this, they stuck needles into their tongues and cheeks. This phenomenon suggests that meditation may affect higher functions of the central nervous system, and indicates the existence of specific neural mechanisms to inhibit or relief the perception of pain through long-term meditation training. Several studies using psychological methods demonstrated that meditators had lower pain sensitivity and experienced analgesic effects during mindful states (Grant and Rainville, 2009; Perlman et al., 2010; Zeidan et al., 2010). Recent studies have attempted to clarify this by using neuroimaging methods, such as functional magnetic resonance imaging (fMRI), and neurophysiological methods, such as magnetoencephalography (MEG) and electroencephalography (EEG) (Kakigi et al., 2005a; Orme-Johnson et al., 2006; Brown and Jones, 2010; Grant et al., 2011; Zeidan et al., 2011; Gard et al., 2012; Lutz et al., 2013; Villemure et al., 2014). These studies provided evidence of modulation regarding pain-related neural activity during meditation. However, the characteristics of this modulation were not consistent among these studies, which will be introduced in Section “Neural Activity of Pain Perception during Meditation” in detail. Thus, these neural mechanisms have been considered as the ‘mystery of meditation.’

In this review, we attempted to elucidate the neural mechanisms involved in the effects of meditation on human pain perception based on published findings with neuroimaging and neurophysiology. To the best of our knowledge, there have been few systematic reviews in the scientific literature on this topic, even though many research reviews have focused on the neural mechanisms of pain perception (Kakigi et al., 2003, 2005b; Apkarian et al., 2005; Tracey and Mantyh, 2007; Wiech et al., 2008b; Inui and Kakigi, 2012; Garcia-Larrea and Peyron, 2013; Moayedi and Davis, 2013) and meditation (Cahn and Polich, 2006; Lutz et al., 2008; Tang and Posner, 2013a,b; Marchand, 2014). Zeidan et al. (2012) and Grant (2014) recently published a review on the brain mechanisms involved in pain relief during meditation. They discussed the underlying mechanisms based on their previous findings and other fMRI studies; however, we considered that additional standpoints were needed for a better understanding. We firstly overviewed brain regions during meditation and the anatomical changes that occurred in the brain with long-term meditation training. We then reviewed the findings of previous studies that examined pain-related activity during meditation. We also described the brain mechanisms responsible for pain relief during meditation, and the reason for paradoxical evidence among previous studies. These processes and ideas were not accessed in previous reviews by Zeidan et al. (2012) and Grant (2014). We believed that the reappraisal of this effect may be associated with an understanding of the mechanisms underlying sensory, cognitive, or affective pain modulation along the lines of perceived control over pain.

Regarding the methodology of this research, we performed several PubMed searches with terms including meditation, mindfulness, pain, fMRI, EEG, MEG, VBM, DTI, ERP, voxel-based morphometry, distraction, placebo, review, electroencephalography, magnetoencephalography, diffusion tensor imaging, and event-related potentials.

## NEURAL ACTIVITY OF PAIN PROCESSING

Pain is a conscious experience, an interpretation of the nociceptive input influenced by memories, as well as emotional, pathological, genetic, and cognitive factors (Tracey and Mantyh, 2007). Thus, the central nervous system in the human brain possesses complex pain-related neural networks, involving sensory-discriminative, affective-motivational, and cognitive-evaluative components, at least partially dissociable in terms of the underlying neural networks (Apkarian et al., 2005; Grant et al., 2011). Neuroimaging and neurophysiological studies provided evidence for these neural networks, which include multiple brain regions often referred to as the ‘pain matrix.’ The pain matrix generally includes the primary somatosensory cortex (SI) contralateral to side of the stimulation, as well as the bilateral secondary somatosensory cortex (SII), insula, anterior cingulate cortex (ACC), and thalamus. The prefrontal cortex (PFC) has been linked to the evaluation and anticipation of pain sensations (Coghill et al., 1999). Previous studies defined the pain matrix as including two systems: lateral and medial systems (see the review by Treede et al., 1999). The lateral system is projected to the cortical level with SI, SII, and the thalamus and is involved in the sensory-discriminative component of pain (stimulus localization, intensity discrimination, and quality discrimination). The medial system involves the ACC and insula, and is associated with the affective-motivational component, which is an essential part of pain (emotional reactions, arousal, attention to the pain stimulation, and escape response). Moreover, recent studies have suggested the existence of a descending pain modulation system, which includes activities in the PFC, ACC, and periaqueductal gray (PAG) of the brainstem (see reviews, Tracey and Mantyh, 2007; Wiech et al., 2008b). Afferent signals related to noxious stimuli are attenuated by the release of opioids from the PAG.

## BRAIN REGIONS DURING MEDITATION

### BRAIN REGIONS DURING MEDITATION

Based on the findings of studies conducted over the last three decades, meditation practices have been shown to have many ameliorative effects on the symptoms of disorders, including anxiety, depression, substance abuse, eating disorders, and chronic pain, and also improve well-being and quality of life (Kabat-Zinn et al., 1992; Teasdale et al., 2000; Hölzel et al., 2011). Meditation is also known to have lasting effects on respiratory control, including respiration rate and oxygen consumption, and the long-term practice of such forms of meditation may induce structural changes in brain regions involved in basic autonomic regulation (Vestergaard-Poulsen et al., 2009). A number of studies investigated brain activation during meditation by utilizing neurophysiological and neuroimaging methods in order to clarify the neural mechanisms during meditation. The characteristics of background EEG activities have often been examined during meditation (for a review, see Cahn and Polich, 2006). One characteristic is the increase of theta and alpha activation associated with proficiency in the meditative technique. Frontal midline activity is generated from the ACC, medial prefrontal cortex (mPFC), and dorsolateral prefrontal cortex (DLPFC; Asada et al., 1999; Ishii et al., 1999), and the activity is correlated with a continuous concentration of attention. Neuroimaging methods including fMRI, PET, and single photon emission computed tomography (SPECT) revealed the brain regions activated during meditation (for a review, see Cahn and Polich, 2006). fMRI studies reported significant activation in the inferior frontal gyrus (IFG), mPFC, ACC, posterior cingulate cortex (PCC), basal ganglia, supplementary motor area (SMA), precentral gyrus, midbrain, inferior parietal lobule (IPL), precuneus, and hippocampus (Lazar et al., 2000; Hölzel et al., 2007; Baerentsen et al., 2010; Davanger et al., 2010; Engström et al., 2010; Ives-Deliperi et al., 2011), while deactivation was observed in the primary (VI) and secondary (VII) visual cortices, PFC, precuneus, insula, ACC, PCC, and occipito-parieto-temporal area (Herzog et al., 1990; Baerentsen et al., 2010; Ives-Deliperi et al., 2011). Newberg et al. (2001) using SPECT showed that regional cerebral blood flow was significantly increased in the cingulate gyrus, inferior, and orbital frontal cortex, DLPFC, and thalamus. Yamamoto et al. (2006) using MEG and EEG simultaneously identified the source of alpha activity in eight meditators during meditation, and localized dipole sources in both the mPFC and ACC.

Sperduti et al. (2012), using a meta-analysis of previous neuroimaging studies on brain regions during meditation, recently reported the core cortical system shared by different kinds of meditation. Prominent activations were detected in the parahippocampal formation, basal ganglia, and mPFC. They hypothesized that the parahippocampal formation evaluated and controlled the stream of mental scenes (thoughts monitoring system), and also that the basal ganglia were responsible for the onset and maintenance of a background attentional set characterized by lower distractibility from irrelevant stimuli (interference control system). They also suggested that mPFC supported the inner engagement of attention and enhanced self-awareness (self-monitoring system).

### BRAIN ACTIVITIES IN DIFFERENT FORMS OF MEDITATION

There are two broad categories of meditation styles; focused attention (FA) meditation and open monitoring (OM) meditation (for a review, see Lutz et al., 2008), involving different attentional, cognitive monitoring, and awareness processes. Lutz et al. (2008) reported that FA meditation entailed the voluntary focusing of attention on a chosen object. The characteristics of this type involve directing and sustaining attention on a selected object (e.g., breath sensation), detecting mind wandering and distractors (e.g., thoughts), disengagement of attention from distractors and shifting attention back to the selected object, and cognitive reappraisal of the distractor (e.g., ‘just a thought,’ ‘it is okay to be distracted’). On the other hand, OM meditation reflects non-reactive monitoring of the content of experience from one moment to the next. This includes no explicit focus on objects, non-reactive meta-cognitive monitoring (e.g., for novices, the labeling of experience), and non-reactive awareness of automatic cognitive and emotional interpretations of sensory, perceptual, and endogenous stimuli.

According to the findings of Lou et al. (1999), the activity pattern of meditation differed according to the meditative content. For example, meditation on the sensations of the weight of limbs and other body parts, presumably related to motor attention, was mainly supported by parietal and superior frontal activities, abstract sensations of joy by left hemisphere parietal and superior temporal (i.e., Wernicke area) activities, and visual imagery by strong activation of the occipital lobe, with sparing of the VI region, and the parietal lobe. Meditation on symbolic representation of the self was supported by bilateral parietal activity.

Shimomura et al. (2008) identified differences in the activated regions of BOLD signals between the Nembutsu, which voices the hope of rebirth into Amida’s Pure Land in the Buddhists, and Sutra. The task of repeating the Nembutsu activated the superior/medial frontal gyrus, while the task of reciting the Sutra activated the lateral middle frontal gyrus, angular gyrus, and supramarginal gyrus.

Manna et al. (2010) compared brain activities between FA and OM meditations within the same subjects. They detected larger activated regions in the DLPFC, precuneus, superior parietal lobule (SPL), insula, IFG, and transverse temporal gyrus during OM meditation than FA meditation, while larger activated regions were observed in the mPFC and ACC during FA meditation than OM meditation.

Wang et al. (2011) also reported differences in brain activation between focused-based and breath-based practices. They demonstrated that the breath-based practice activated many regions in the hippocampus, parahippocampus, amygdala, insula, IFG, superior temporal gyrus (STG), and fusiform gyrus, whereas focused-based practice was associated more closely with activation of the precentral gyrus, precuneus, and insula.

These studies using neuroimaging methods suggested the existence of different neural networks depending on the style of meditation. Based on these findings, OM meditation may activate larger brain regions including the limbic system than FA meditation.

## NEUROPLASTICITY OF BRAIN STRUCTURES WITH LONG-TERM MEDITATION

Several studies focused on the neuroplasticity of brain structures in relation to the long-term practice of meditation. One approach used to analyze neuroplasticity was comparing the thickness of the cerebral cortex between meditators and non-meditators, with an anatomical MRI scan (Fischl and Dale, 2000). The cortical thickness is estimated as the distance between the gray/white boundary and outer cortical surface. The segmentation procedure uses both intensity and continuity information from three-dimensional high-resolution MRI, including the coronal, axial, and sagittal planes (Salat et al., 2004). A second approach was voxel-based morphometry (VBM). VBM has frequently been used to clarify differences in the gray matter (GM) between different subject groups, as well as changes in GM within the same subject between pre- and post-training (i.e., longitudinal study). For example, this method has been utilized to examine the long-term effects of training on the volumes of GM, such as juggling skills (Draganski et al., 2004; Boyke et al., 2008; Scholz et al., 2009), golf (Bezzola et al., 2011), large-scale space (Maguire et al., 2000), bilingualism (Mechelli et al., 2004), arithmetic (Aydin et al., 2007), music (Han et al., 2009; Hyde et al., 2009), and revising for exams (Draganski et al., 2006). In a study by Draganski et al. (2004) involving juggling training for 3 months, they showed a transient and selective structural change in brain areas associated with the processing and storage of complex visual motion in the mid-temporal area (hMT/V5) and left posterior intraparietal sulcus. This study suggested that long-term training led to neuroplasticity in human brains, which was related to functional rather than anatomical changes. Thus, it is likely that similar neuroplasticity occurs in meditators on long-term training in meditation. A third approach was diffusion tensor imaging (DTI). DTI has been used to evaluate the anatomical connectivity of white matter (WM) with fractional anisotropy (FA; Taubert et al., 2012). Similar to VBM, DTI has been employed to clarify structural changes in WM with long-term training such as music (Bengtsson et al., 2005; Imfeld et al., 2009; Oechslin et al., 2010) and physical activity (Tseng et al., 2013; Herting et al., 2014). Just like GM, the WM density may also be affected by long-term training in meditation. Indeed, several studies reported that both GM and WM were changed by long-term training, such as in juggling skills (Scholz et al., 2009), suggesting that the WM changes underlie behavioral improvements by altering the conduction velocity and synchronization of nervous signals, which are regulated by myelin.

We briefly introduced previous studies that examined neuroplasticity through long-term meditation training in the next subsection, and these are listed in **Table 1**.

**Table 1:** Brain regions altered by long-term meditation training.

Authors	Measurement	Brain regions altered by long-term meditation training
Lazar et al. (2005)	Cortical thickness	Right AI, right PFC, left STG, and left SI
Pagnoni and Cekic (2007)	VBM	Left putamen
Hölzel et al. (2008)	VBM	Right AI, left ITG, and right hippocampus
Luders et al. (2009)	VBM	Right OFC, right hippocampus, and left ITG
Vestergaard-Poulsen et al. (2009)	VBM	Brainstem
Grant et al. (2010)	Cortical thickness	Bilateral ACC, bilateral SII, bilateral insula, bilateral parahippocampal gyrus, bilateral SI, and bilateral PFC
Hölzel et al. (2010)	VBM	Right amygdala
Tang et al. (2010)	DTI	ACC
Hölzel et al. (2011)	VBM	Left hippocampus, PCC, left TPJ, and the cerebellum
Luders et al. (2011)	DTI	Commissural pathways, association pathways, corticospinal tract, temporal component of superior longitudinal fasciculus, and uncinate fasciculus
Tang et al. (2012)	DTI	Genu and body of the corpus callosum, bilateral corona radiata, left anterior corona radiata, and left superior longitudinal fasiculus
Luders et al. (2012a)	DTI and MRI	Corpus callosum
Luders et al. (2012b)	Gyrification	Bilateral AI, left pre/postcentral gyrus, left central sulcus, left ITG, left angular gyrus, left parieto-occipital fissure, right parietal operculum, right fusiform gyrus, and right cuneus
Murakami et al. (2012)	VBM	Right AI, and right amygdala
Luders et al. (2013a)	MRI volume	Bilateral hippocampi
Luders et al. (2013b)	VBM	Bilateral hippocampi
Kang et al. (2013)	Cortical thickness	Bilateral MTG/ITG, bilateral OFC, bilateral PFC, bilateral superior frontal cortex, bilateral PPC, right PCC, right cuneus, right fusiform gyrus
	DTI	Bilateral cuneus, bilateral ITG, bilateral temporal pole, left forceps minor, left frontal pole, left precuneus, left lateral occipital cortex, left precental cortex, left parahippocampal gyrus, left PCC, right insula, right ACC, right subcallosal cortex, right superior corona radiation, right OFC, and right anterior thalamic radiation
Leung et al. (2013)	VBM	Right angular gyrus, right parahippocampal gyrus, left ITG, and left MTG
Pickut et al. (2013)	VBM	Bilateral TPJ, left lingual gyrus, left cuneus, left thalamus; bilateral hippocampi, right amygdala, and bilateral caudate nucleus
Wells et al. (2013)	Connectivity	Connectivity between PCC and bilateral PFC, and between PCC and left hippocampus
	MRI volume	Bilateral hippocampi
Kurth et al. (2014)	VBM	Superior parietal lobule
Singleton et al. (2014)	VBM	Brainstem

*VBM, voxel-based morphometry; DTI, Diffusion tensor imaging; AI, anterior insula; PFC, prefrontal cortex; STG, superior temporal gyrus; SI, primary somatosensory cortex; ITG, inferior temporal gyrus; OFC, orbito-frontal cortex; ACC, anterior cingulate cortex; SII, secondary somatosensory cortex; PCC, posterior cingulate cortex; TPJ, temporo-parietal junction; MTG, middle temporal gyrus; PPC, posterior parietal cortex.*

### CORTICAL THICKNESS AND MRI VOLUME

Lazar et al. (2005) compared cortical thicknesses between 20 participants with extensive training in insight meditation and 15 control participants. The right anterior insula (AI), right PFC, left STG, and left SI were significantly thicker in meditators than in controls.

Grant et al. (2010) investigated cortical thicknesses in 19 Zen meditators and 20 controls. Meditators were found to have a thicker cortex in the bilateral ACC, bilateral SII, bilateral insula, bilateral parahippocampal gyrus, bilateral SI, and bilateral PFC. Furthermore, when assessed in all subjects, lower pain sensitivity was associated with a thicker cortex in affective, pain-related regions including the ACC, bilateral parahippocampal gyrus, and AI.

Kang et al. (2013) employed a whole-brain cortical thickness analysis in the brains of 46 experienced meditators and 46 matched control volunteers. Cortical thickness was significantly greater in meditators than in controls in the bilateral middle temporal gyrus (MTG)/inferior temporal gyrus (ITG), bilateral orbito-frontal cortex (OFC), bilateral PFC, bilateral superior frontal cortex, bilateral posterior parietal cortex (PPC), right PCC, right cuneus, and right fusiform gyrus.

In a study performed by Luders et al. (2013a), high-resolution structural MRI data from 30 long-term meditators and 30 controls were analyzed to explore hippocampal features in the framework of meditations. Bilateral hippocampal volumes were larger in meditators than in controls, and significantly so for the left hippocampus.

Wells et al. (2013) randomized 14 adults with mild cognitive impairments into mindfulness-based stress reduction (MBSR) or usual care interventions, and recorded brain morphometry before and after the intervention. MBSR participants had higher functional connectivity between the PCC, bilateral mPFC, and left hippocampus than that of the controls. MBSR participants also had trends of less bilateral hippocampal volume atrophy than the controls.

These studies indicated that long-term meditation increased cortical thickness in several brain regions, such as SI, SII, PFC, temporal gyrus, PPC, ACC, and hippocampus, and the connectivity was also enhanced among PCC, PFC, and hippocampus.

### VOXEL-BASED MORPHOMETRY

Pagnoni and Cekic (2007) employed VBM in 13 regular practitioners of Zen meditation and 13 matched controls, and a significant difference was observed in GM in the left putamen.

Hölzel et al. (2008) investigated the MRI brain images of 20 mindfulness (Vipassana) meditators and compared regional GM concentrations to those of non-meditators. The findings obtained revealed greater GM concentrations for meditators in the right AI, left ITG, and right hippocampus.

Luders et al. (2009) detected significantly larger GM volumes in the right OFC, right hippocampus, and left ITG in 22 meditators than in 22 controls.

Vestergaard-Poulsen et al. (2009) showed that there were structural differences in the brainstem regions, which were associated with cardiorespiratory control, between 10 long-term practitioners of meditation and 10 controls.

Hölzel et al. (2010) conducted a longitudinal MRI study to investigate the relationship between changes in perceived stress with changes in amygdala GM density following 8 weeks of MBSR. Following the intervention, participants reported significantly reduced perceived stress, and reductions in perceived stress positively correlated with decreases in right amygdala GM density.

Hölzel et al. (2011) performed a longitudinal study on 16 meditation naïve participants to investigate pre–post changes in brain GM concentrations attributable to participation in an 8-week MBSR program. The VBM analysis showed increases in GM concentration within the left hippocampus, PCC, left temporo-parietal junction (TPJ), and the cerebellum.

Murakami et al. (2012) applied VBM to investigate the relationship between brain structures and each facet as measured by the Five Facet Mindfulness Questionnaire (FFMQ). The findings obtained showed a positive association between the describing facet of mindfulness on the FFMQ and GM volume in the right AI and right amygdala.

Luders et al. (2013b) examined GM characteristics in a large sample of 100 subjects (50 meditators and 50 controls), in which meditators had been practicing for approximately 20 years on average. A standard, whole-brain VBM approach was applied and revealed significant meditation effects in the vicinity of the hippocampus, showing that the volume of GM was greater in meditators than in controls as well as positive correlations with the number of years practiced.

Leung et al. (2013) demonstrated that the GM volume was greater in the right angular gyrus, right parahippocampal gyrus, left ITG, and left MTG in experts of loving-kindness meditation, than in controls.

Pickut et al. (2013) compared differences in the GM density between 14 patients with Parkinson’s Disease (PD) following an 8-week MBSR and 13 patients with PD following usual care. GM density was higher in the MBSR group than in the usual care group in the bilateral TPJ, left lingual gyrus, left cuneus, left thalamus, bilateral hippocampi, right amygdala, and bilateral caudate nucleus.

Kurth et al. (2014) investigated differences in GM asymmetry as well as correlations between GM asymmetry and years of meditation practice in 50 long-term meditators and 50 controls. They observed significant differences between meditators and controls with respect to GM asymmetry in the SPL.

Singleton et al. (2014) showed that the scores on five psychological well-being (PWB) subscales as well as the PWB total score increased significantly over the MBSR course. In addition, these changes positively correlated with GM concentration increases in two symmetrically bilateral clusters in the brainstem, which contained the area of the pontine tegmentum, locus coeruleus, nucleus raphe pontis, and sensory trigeminal nucleus.

Luders et al. (2012b) examined cortical gyrification in a large sample (*n* = 100) of meditators and controls. Cortical gyrification was established by calculating the mean curvature across 1000s of vertices on individual cortical surface models. Gyrification in meditators was prominent within the bilateral AI, left pre/postcentral gyrus, left central sulcus, left ITG, left angular gyrus, left parieto-occipital fissure, right parietal operculum, right fusiform gyrus, and right cuneus.

As shown in VBM studies, long-term meditation increased the GM volume in the OFC, PPC, temporal gyrus, lingual gyrus, cuneus, thalamus, insula, PCC, cerebellum, hippocampus, amygdala, basal ganglia, and brainstem. Thus, GM in both the cerebral cortex and the limbic system may be larger in meditators than in controls.

### DIFFUSION TENSOR IMAGING

Tang et al. (2010) examined all brain areas showing FA changes between pre- and post-training with 11 h of integrative body–mind training. They demonstrated significantly greater FA in the anterior corona radiata associated with the ACC.

Luders et al. (2011) investigated WM fiber characteristics in a well-matched sample of long-term meditators and controls. The findings obtained showed more pronounced structural connectivity in meditators than in controls throughout the commissural pathways, association pathways, corticospinal tract, temporal component of the superior longitudinal fasciculus, and uncinate fasciculus.

Luders et al. (2012a) analyzed differences in the corpus callosum between 30 meditators and 30 controls. Callosal measures, particularly in anterior callosal sections, were larger in long-term meditators than in controls, indicating the greater connectivity of hemispheric integration involving prefrontal regions.

Tang et al. (2012) reported different patterns of FA increases in brain regions following 4 weeks of integrative body–mind training. The first pattern was a decrease in both radial diffusivity (RD) and axial diffusivity (AD) accompanied by increased FA, and occurred in six brain regions involving the genu and body of the corpus callosum, bilateral corona radiata, left anterior corona radiata, and left superior longitudinal fasiculus. The second pattern was a decrease in RD accompanied by increased FA, and was detected in the body of the corpus callosum, left corona radiata, left anterior corona radiata, and left superior longitudinal fasiculus.

In addition to whole-brain cortical thickness analyzes, Kang et al. (2013) used DTI. Direct comparisons of FA values between meditators and controls revealed significant differences in most of the anterior portion of the brain, with higher FA values in the cuneus, precuneus, and occipital regions as well as the ventromedial PFC in the meditator group. In contrast, meditators showed lower FA values in the right MPFC, PCC, and right occipital regions than those in the controls.

These previous findings using the DTI method revealed that long-term meditation enhanced the anatomical connectivity of WM including the corona radiata, superior longitudinal fasciculus, and uncinate fasciculus, and corpus callosum.

## NEURAL ACTIVITY OF PAIN PERCEPTION DURING MEDITATION

As described in the Section “Introduction,” recent studies using neuroimaging and neurophysiological methods demonstrated that meditation reduced pain perception. We have introduced these studies in this section. We initially considered why meditation modulated pain perception. Previous studies reported the modulation of pain perception and pain-related neural activities during distraction (Yamasaki et al., 1999, 2000; Qiu et al., 2004), placebo (Petrovic et al., 2002; Wager et al., 2004), voluntary movements (Kakigi and Shibasaki, 1992; Kakigi et al., 1993; Nakata et al., 2004, 2009), odors (Villemure et al., 2003), emotion (Roy et al., 2009), and religious contemplation (Wiech et al., 2008a). We suggested three possible hypotheses; (1) an attention effect (distraction away from pain itself); (2) placebo effect; and (3) modulation of neural activity in the ‘pain matrix.’ Regarding hypothesis 1, MEG and EEG studies previously showed that brain activity relating to pain perception decreased during distraction tasks, and linked processes with attentional control. For example, Yamasaki et al. (1999) reported that MEG responses that peaked approximately 160 ms after a painful CO_2_ laser stimulation to the right forearm were not affected during a distraction task (calculating), whereas sequential EEG responses that peaked at 240–340 ms were markedly affected. They also confirmed this effect with MEG and EEG by using a painful electrical stimulation to the right index finger (Yamasaki et al., 2000). Qiu et al. (2004) also recorded MEG and EEG following the stimulation of a small surface area with a CO_2_ laser, which elicited brain activity for the selective activation of the C afferent sensory terminal, and compared activities between resting control and distraction tasks (calculating). In that study, the strength of equivalent current dipoles (ECDs) in six brain regions, which included the SI contralateral to the side of the stimulation, bilateral SII, bilateral the medial temporal area, and the cingulate, was significantly smaller during the distraction task than during the resting control task. These studies using MEG and EEG indicated that the strength of neural activities relating to pain perception decreased during distraction tasks. In addition to the distraction effect, the placebo effect on pain perception has been examined, and is often referred to as placebo analgesia. Previous reviews described the neural mechanisms underlying placebo analgesia (Wiech et al., 2008b; Qiu et al., 2009), and indicated that pain-related neural activity, such as that in the thalamus, ACC, and insula, decreased during the placebo condition, while the magnitudes of these decreases correlated with subjective pain relief afforded by the placebo. Prefrontal activation has also been observed prior to a noxious stimulation, indicating an interplay between expectations and reappraisal in the placebo effect (Wiech et al., 2008b). A stereotypical image regarding meditation already exists in participants before experiments are performed. This state should be associated with a similar effect to that of placebo analgesia. However, we speculated whether the effects of meditation on pain perception could be simply explained by the distraction and placebo effects. We subsequently considered specific neural mechanisms; the modulation of neural activity for the ‘pain matrix’ during meditation. Some published findings supported this notion.

### PREVIOUS FINDINGS USING NEUROPHYSIOLOGICAL METHODS

**FIGURE 1:**
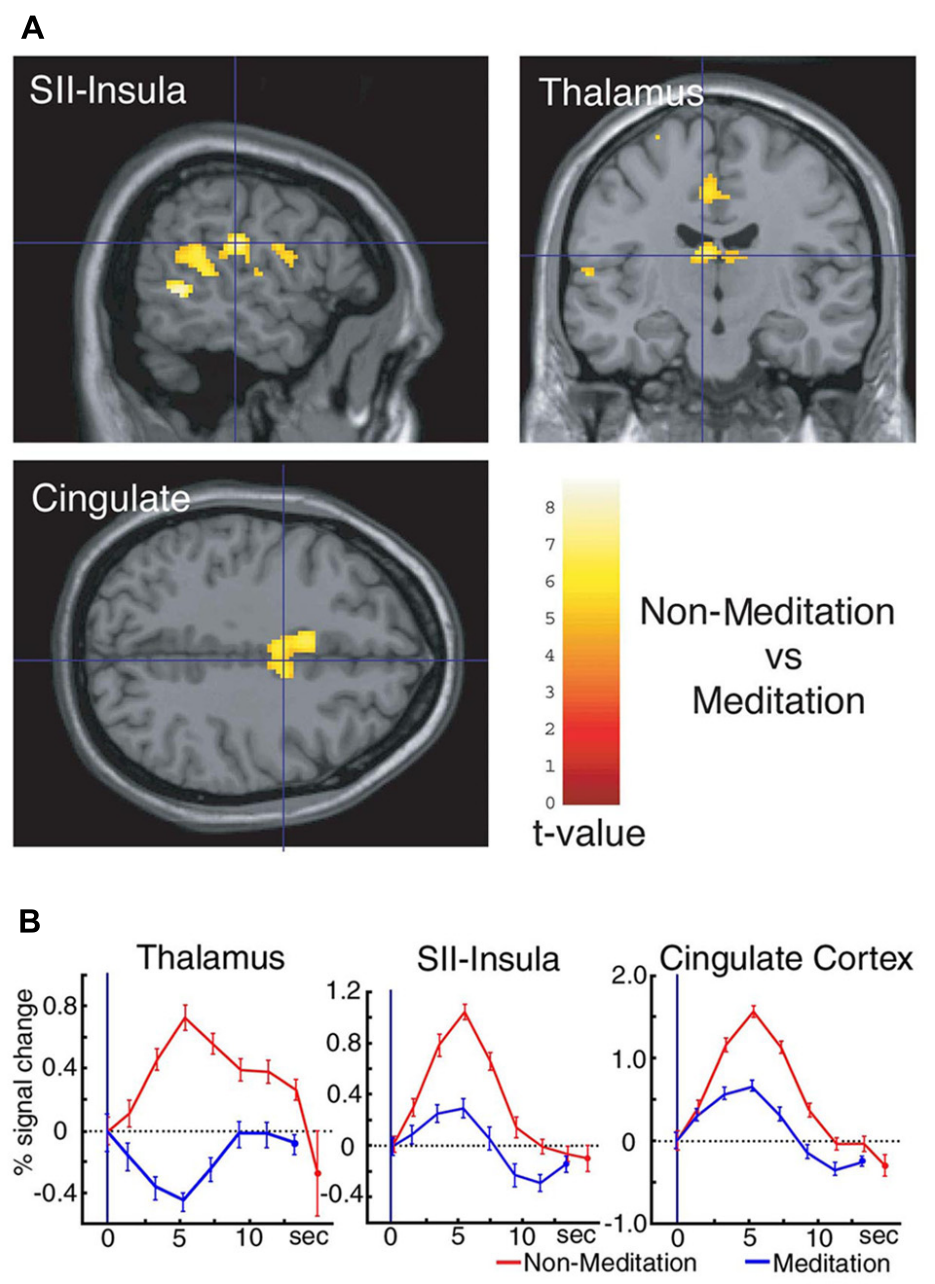
**(A)** Significant differences were observed between non-meditation and meditation in three regions, the thalamus, SII-insula, and cingulate cortex. **(B)** Time course of the hemodynamic response (HDR) in the thalamus, SII-insula, and cingulate cortex during both non-meditation and meditation in the Yoga Master following a noxious YAG laser stimulation applied to the dorsum of the left foot. Activities in these regions decreased during meditation, and levels were lower than the baseline in the thalamus. Error bars indicate the SD of trials. Adopted from Kakigi et al. (2005a).

The first study using neuroimaging and neurophysiological methods to show the effects of meditation on pain perception was ours. We recorded brain activity relating to pain perception in a Yoga master by utilizing MEG and fMRI, and compared differences in this activity between meditation and non-meditation (Kakigi et al., 2005a). This study consisted of only one person, and, as such, was a case report for the effects of meditation on pain perception. The Yoga master that participated in this study was very special because he had achieved the title of Yoga Samrat, indicating the highest level of proficiency, from the Indian Yoga Culture Federation in 1983. We identified several characteristics during meditation. We analyzed background MEG activity, and found that the power of alpha frequency bands peaking at approximately 10 Hz was larger during meditation than non-meditation in the occipital, parietal, and temporal regions. Cortical activities in the SI and SII detected by MEG were also very weak or absent during meditation following a painful laser stimulation applied to the dorsum of the left hand. BOLD signals in fMRI data revealed marked changes in the levels of neural activities in the thalamus, SII-insula, and cingulate cortex between meditation and non-meditation. Activities in these regions increased during non-meditation, which was similar to that observed in normal subjects. On the other hand, these activities decreased during meditation (**Figure 1**). In addition, activities in other regions such as frontal lobe, parietal lobe, and midbrain involving PAG increased during meditation. This study revealed marked changes in multiple regions related to pain perception during meditation.

Brown and Jones (2010) compared event-related potentials (ERPs) related to anticipatory processing before pain stimuli and pain processing after the stimulation between meditator and control groups. More experienced meditators perceived the pain as being less unpleasant than the controls, with meditation experience correlating inversely with unpleasantness ratings. The amplitude of ERPs for anticipatory processing was significantly smaller in the meditator group than in the control group, whereas no significant difference was observed in the peak amplitude of the P2 component for pain processing between groups. Low-resolution electromagnetic tomography (LORETA), which was performed to analyze the ERP source for anticipation, showed that activation of the mid-cingulate cortex (MCC) and inferior parietal cortex during late anticipation was lower in meditator group than in the control group. In addition, activity in the MCC was positively correlated with pain unpleasantness in the meditator group, but not in the control group. Activity in the ventromedial-PFC (vmPFC) correlated negatively with pain unpleasantness in the meditator group and positively in the control group. Activity in the SII and posterior insula (PI) after the pain stimulation was lower during meditation, in spite of no significant difference in the peak amplitude of P2. These findings indicated that meditation reduced anticipation for the pain stimulation; however, its effects on pain-evoked activity were less clear.

### PREVIOUS FINDINGS USING NEUROIMAGING METHODS

Orme-Johnson et al. (2006) recorded fMRI responses to thermal pain stimuli, and used two groups for the experiments. One group comprised healthy control subjects, who had trained in meditation for 5 months. fMRI was recorded pre- and post training. The other group included age-matched healthy long-term meditators who had practiced meditation techniques for a mean of approximately 31 years. In the pre-training period, the long-term meditators showed 40–50% lower responses than the healthy controls in the thalamus, PFC, and ACC for the thermal pain stimulation. Moreover, after learning and practicing meditation techniques for 5 months, brain responses in the healthy control group decreased by 40–50%, with no significant changes being observed in the long-term meditators. All fMRI recordings in their study were conducted outside the meditation period, not during it. Thus, distractions from pain by meditation were not relevant. One limitation of this study was that they only investigated neural activities in the thalamus, PFC, and ACC, and not in other brain regions such as the SI, SII, insula, and amygdala.

Grant et al. (2011) used a thermal stimulator to induce pain, and compared pain-related activity with fMRI between Zen meditators and controls. Neural activity in the PFC, amygdala, and hippocampus was decreased, whereas activation of the ACC, thalamus, and insula was increased in Zen meditators. In contrast, activation of the DLPFC, amygdala, middle frontal gyrus, hippocampus, and med-PFC/orbitofrontal cortex (OFC) was stronger in the controls. However, paradoxically, a correlation was noted between pain-related activation and meditation experience. In other words, the most experienced practitioners showed lower responses in the ACC, thalamus, and insula, which was consistent with the findings of Kakigi et al. (2005a).

Zeidan et al. (2011) also investigated pain-related brain activity using fMRI before and after meditation training for 4 days. They showed that meditation reduced pain-related activation of the SI contralateral to the thermal pain stimulation, which was a region associated with the sensory-discriminative processing of nociceptive information (Coghill et al., 1999). Meditation-induced reductions in pain intensity ratings were associated with increased activity in the ACC and AI. Reductions in pain unpleasantness ratings were associated with OFC activation and thalamic deactivation. These findings suggested that meditation modulated multiple brain activities that alter the construction of the subjectively available pain experience from afferent information.

Gard et al. (2012) recorded fMRI in meditation practitioners and control subjects during meditation and mindfulness and control conditions following unpleasant electric stimuli. Pain unpleasantness was 22% less and anticipatory anxiety was 29% less in meditation practitioners during meditation, but not in control subjects. This reduction was associated with decreased activation of the PFC and increased activation of the right PI during the stimulation, as well as increased activation of the ACC during the anticipation of pain.

Lutz et al. (2013) used fMRI and detected differences in neural activation patterns associated with pain, its anticipation, and habituation between expert meditators and novices. Three main findings were reported: Expert practitioners gave lower unpleasantness ratings, and had stronger BOLD activity in the left AI and MCC during pain, than novices. Experts had less anxiety-related baseline activity in these clusters and in the amygdala prior to pain. Experts had faster neural habituation to pain and its anticipation; the smaller the anticipatory activity in the amygdala, the faster the neural habituation in response to pain in the MCC.

### RELATIONSHIP BETWEEN BRAIN STRUCTURE AND PAIN PERCEPTION

Villemure et al. (2014) investigated thermal detection and pain thresholds as well as cold pain tolerance in experienced North American Yoga practitioners and controls subjects. To clarify the underlying neuroanatomical mechanisms of perceptual changes, they also examined structural differences in brain GM and WM between the yogis and controls by using VBM in MRI recording. They found that insular GM correlated with pain tolerance. Insular GM volumes in yogis positively correlated with Yoga experience, suggesting a causal relationship between Yoga and insular size. Yogis also had higher left intrainsular WM integrity. These findings suggested that the regular and long-term practice of Yoga improved pain tolerance by changing insular brain anatomy and connectivity.

The pain-related brain regions modulated by meditation are listed in **Table 2**.

**Table 2:** Pain-related brain regions affected by meditation.

Authors	Effects of meditation on brain activity
Kakigi et al. (2005a)	Increase: left SMI, right SPL, right DLPFC, bilateral SFG, and bilateral midbrain
	Decrease: bilateral thalamus, bilateral SII-insula, and cingulate cortex
Orme-Johnson et al. (2006)	Increase: absent
	Decrease: thalamus, PFC, and ACC
Brown and Jones (2010)	Increase: absent
	Decrease: left MCC, right IPL, right SII, and left PI
Grant et al. (2011)	Increase: bilateral ACC, bilateral thalamus, left insula, and left SII
	Decrease: bilateral DLPFC, bilateral amygdala, right OFC, and right hippocampus
Zeidan et al. (2011)	Increase: ACC, right AI, and OFC
	Decrease: SI and thalamus
Gard et al. (2012)	Increase: right PI and ACC
	Decrease: PFC, cerebellum, and STG
Lutz et al. (2013)	Increase: left AI and MCC
	Decrease: amygdala

*SMI, primary sensorimotor cortex; SPL, superior parietal lobule; DLPFC, dorsolateral prefrontal cortex; SFG, superior fronal gyrus; SII, secondary somatosensory cortex; ACC, anterior cingulate cortex; MCC, midcingulate cortex; IPL, inferior parietal lobule; PI, posterior insula; SI, primary somatosensory cortex; AI, anterior insula; OFC, orbito-frontal cortex; STG, superior temporal gyrus.*

## DISCUSSION

A review of previous studies using EEG, MEG, and fMRI on the effects of meditation effect on pain-related neural activity revealed difficulties in interpreting their findings due to paradoxical evidence. Some studies reported increases in neural responses to pain stimuli during meditation in the ACC and insula (Grant et al., 2011; Zeidan et al., 2011; Gard et al., 2012; Lutz et al., 2013), whereas others showed a decrease (Kakigi et al., 2005a; Orme-Johnson et al., 2006; Brown and Jones, 2010; **Table 2**). In general, since the activities of the ACC and AI were correlated with pain perception (Wiech et al., 2008b), increases in neural activities during meditation may be related to enhancements rather than reductions in pain perception. This contradiction may directly contribute to the ‘mystery of meditation,’ and we proposed two possible hypotheses.

### HYPOTHESES

One was the number of years of experience of the meditation. Grant et al. (2011) reported difference in the strength of neural activities in the ACC, DLPFC, and mPFC/OFC that were dependent on the number of years of experience of the meditation. Interestingly, pain activation in the ACC and insula was lower with higher meditation experience. In the study conducted by Kakigi et al. (2005a), as described above, a Yoga master who had achieved the highest level of proficiency had lower pain-related neural activity in the thalamus, SII-insula, and cingulate cortex. That is, the neural activities through long-term training might be related to its spent time. For example, 3 weeks of daily practice on finger movements evoked significantly larger activation of the MI than the control, untrained sequence (Karni et al., 1998). However, long-term physical training (e.g., 10 years) may evoke decreases in neural activities in the MI rather than increases. Such a phenomenon has been reported in musicians and athletes, who often started training very early in childhood, throughout their entire careers (Münte et al., 2002; Nakata et al., 2010), and has frequently been referred to as neural efficiency (Del Percio et al., 2008). Based on the theory of motor learning and neural efficiency, the strength of the neural response in the ACC and insula to pain stimulation during meditation may reflect the proficiency of meditators, even if the unpleasantness of pain and anticipatory anxiety were lower in meditators in novices.

The second hypothesis was the kind of meditation. There are several meditation methods, such as Yoga, Zen, mindfulness of breathing, and Samatha. Thus, differences in the meditation style may be associated with pain-related brain responses. As described in Section “Brain Regions during Meditation,” there are two styles of meditation: FA and OM meditation. Perlman et al. (2010) published behavioral findings from a comparison between novice and long-term meditation practitioners using these techniques. Self-reported unpleasantness, but not the intensity, of painful stimuli while practicing OM were significantly lower in long-term meditators than in novices. No significant effects were found for FA.

Meditation may mainly affect the medial system of pain perception, which involves the ACC, and insula, even though activation or deactivation occurred. Based on the findings of Zeidan et al. (2011), since meditation-induced changes in SI did not specifically correlate with reductions in either pain intensity or unpleasantness, meditation did not affect the lateral system in the SI. On the other hand, pain-related activity in the SII, which belongs to the lateral system, may be affected by meditation. Some studies found stronger activation of the SII during meditation (Grant et al., 2011), while others observed its deactivation (Kakigi et al., 2005a; Brown and Jones, 2010). The modulation of neural activity in the SII may be similar to that in the ACC and insula, even if activation or deactivation occurred. Thus, the effects of meditation on pain processing may be different between the strength of the SI and SII.

Furthermore, regarding activity in the thalamus, three studies showed its deactivation (Kakigi et al., 2005a; Orme-Johnson et al., 2006; Zeidan et al., 2011). Grant et al. (2011) reported that the pain-related neural activity in the thalamus of a Yoga master with the highest level of proficiency was reduced. Deactivation of the thalamus may also be important for understanding the effects of meditation on pain perception, and may be related to a filtering function and the modulation of ascending pain sensory information.

Taking previous findings into consideration, OM meditation rather than FA meditation should attenuate pain-related neural activity in the SII, ACC, insula, and thalamus.

### UNDERLYING MECHANISMS IN MODULATION OF PAIN PERCEPTION

Previous findings revealed increased brain activation in some regions even in meditators with more experience of meditation. For example, Kakigi et al. (2005a) reported that the left foot region of the primary sensorimotor area (SMI), SPL, superior frontal gyrus (SFG), DLPFC, and tectum mesencephali were activated during meditation following laser stimulation of the dorsum of the left foot in a meditator with the highest level of proficiency. The activation of these brain regions was not detected during non-meditation. One possibility for this finding is the specific attention system during meditation. It should be noted that activation was greater in the foot region in SMI. As described in Section “Neural Activity of Pain Perception during Meditation,” if an attention effect and/or placebo effect exists during meditation, activation should be decreased. Moreover, activation was greater in SPL, and SFG/DLPFC, which is consistent with brain regions in the fronto-parietal network related to the attention system (for a review, see Chica et al., 2013). Therefore, we inferred the existence of a specific attention system during meditation that may modulate pain perception during meditation.

In addition to the attention system, meditation affects cognitive control, emotional regulation, cardiorespiratory control, and thinking patterns, because **Table 1** shows many brain regions altered by long-term meditation training. The human brain contains many neural networks, and it may be necessary to consider the functional mechanisms over the simple pain matrix to interpret the ‘mystery of meditation.’ For example, several studies reported that default mode network was changed with long-term meditation training (Taylor et al., 2011; Berkovich-Ohana et al., 2012). The default mode network has been identified to occur during task-induced deactivations, or during brain activity associated with a passive fixation baseline condition related to specific attention-demanding visual tasks (Gusnard and Raichle, 2001; Spreng, 2012). This network involves the medial frontal gyrus, medial and lateral parietal areas, and posterior cingulate (Fox et al., 2005), and is related to active cognitive processing that includes mind-wandering (Mason et al., 2007), and autobiographical remembering (Spreng and Grady, 2010). Thus, modulation in the neural activity of the default mode network with long-term meditation training reflects the changing of automatic processes, such as automatic thoughts, unconscious awareness, and passive attention. Moreover, some studies have also shown that meditation training improves emotional regulation, involving the amygdala (Taylor et al., 2011; Reva et al., 2014). The amygdala function is impaired in many disorders including depression, anxiety, and post-traumatic stress disorders, and attention system and emotional regulation are interactively affected (Desbordes et al., 2012). Therefore, other brain functions including attention, the resting state, and emotional regulation should be related to underlying neural mechanisms for the modulation of pain-related neural activity.

## CONCLUSION

As described in the Section “Introduction,” many studies have examined neural activities associated with pain perception and meditation. However, the neural mechanisms responsible for the effects of meditation have yet to be elucidated in detail. After a thorough literature search, we hypothesized that meditation mainly attenuated the medial system of pain perception including brain regions in the ACC and insula, as well as the lateral system in the SII and thalamus. We speculated that the characteristics of the modulation of this activity depended on the number of years of experience of meditation and/or the kind of meditation, which were associated with paradoxical findings among previous studies that investigated pain-related neural activities during meditation.

In addition, further studies are necessary to improve our understanding of the neurophysiological and psychological mechanisms underlying the effects of meditation on pain perception. For example, the descending pain modulatory system at the level of the brainstem may play an important role in pain relief during modulation (Tracey and Mantyh, 2007; Wiech et al., 2008b). These findings may be related to other neural mechanisms involved in the ‘mystery of meditation.’

## Conflict of Interest Statement

The authors declare that the research was conducted in the absence of any commercial or financial relationships that could be construed as a potential conflict of interest.
